# Supplementary material for: First Genomic Evidence of a Henipa-like Virus in Brazil
Source: Viruses. 2022 Sep 30;14(10):2167. doi: 10.3390/v14102167 (PMC9608811; doi:10.3390/v14102167)
Supplement: Supplementary file 1 [file viruses-14-02167-s001.zip › Supplementary Figure.pdf]

Supplementary Figure

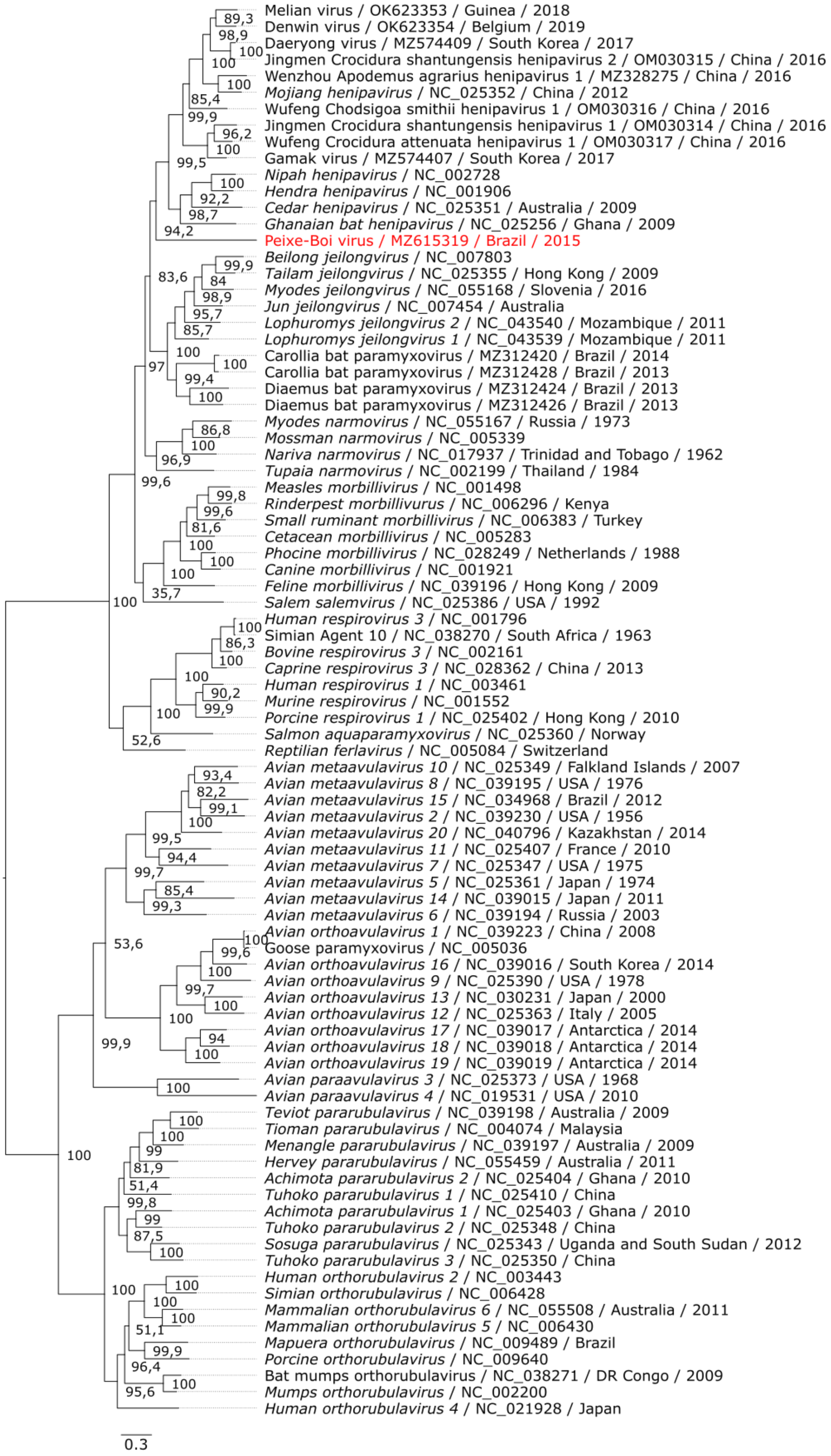

Figure S1. Phylogenetic tree based on the partial nucleotide *Paramyxoviridae* L gene sequences, novel rodent and shrew-borne henipaviruses, Brazilian jeilongviruses, and PBV (in red) sequences. Each record consists of the virus species/name, accession number, country and year of detection/isolation.
